# Kunxian capsules in the treatment of patients with ankylosing spondylitis: a randomized placebo-controlled clinical trial

**DOI:** 10.1186/s13063-016-1438-6

**Published:** 2016-07-22

**Authors:** Qiuxia Li, Li Li, Liqi Bi, Changhong Xiao, Zhiming Lin, Shuangyan Cao, Zetao Liao, Jieruo Gu

**Affiliations:** The Third Affiliated Hospital of Sun Yat-sen University, Guangdong, China; China-Japan Union Hospital of Jilin University, Changchun, China; Integrated Traditional and Western Medicine Hospital of Southern Medical University, Guangzhou, China

**Keywords:** Kunxian capsule, Ankylosing spondylitis, Efficacy, Safety

## Abstract

**Background:**

Ankylosing spondylitis (AS) is a chronic inflammatory autoimmune disease. Kunxian capsule, a Chinese patent medicine which has been used in the treatment of immunologic diseases for many years in China, has anti-inflammatory and immunoregulatory effects. This study investigates the efficacy and safety of Kunxian capsules in the treatment of AS.

**Method:**

This was a randomized, double-blind, parallel control clinical trial involving 80 patients with AS who fulfilled the modified New York criteria (1984) and had active disease defined by a Bath Ankylosing Spondylitis Disease Activity Index (BASDAI) ≥40 mm under background stable nonsteroidal anti-inflammatory drugs (NSAIDs) for more than 4 weeks. Patients were randomly divided into two groups, the Kunxian group and the placebo group, and Kunxian (0.6 g, three times per day) and the placebo were provided for 12 weeks. The primary endpoint was the Assessment of SpondyloArthritis international Society (ASAS) 20 response rate at week 12. The secondary endpoints were ASAS 40, BASDAI 50, the Bath Ankylosing Spondylitis Functional Index (BASFI), Bath Ankylosing Spondylitis Metrology Index (BASMI), and Ankylosing Spondylitis Disease Activity Score on the basis of C-reactive protein level (ASDAS-CRP) at weeks 2, 6, and 12.

**Results:**

The primary endpoint of ASAS 20 at week 12 was achieved in 13 of 35 patients (37.1 %) among the Kunxian group, compared with 4 of 33 (12.1 %) in the placebo group (*p* < 0.05). Significant improvement (BASDAI 50) was also observed between the Kunxian group and the placebo group at week 6 (14 (40 %) and 5 (15.5 %), respectively, *p* < 0.05). At weeks 2, 6, and 12, the ASDAS-CRP level of the Kunxian group was significantly lower than that of the placebo group, especially at week 6 (*p* < 0.01). Kunxian obviously reduced CRP levels compared to placebo at weeks 2, 6, and 12 (*p* < 0.05). Compared with the placebo, Kunxian was associated with greater improvements in signs and symptoms of patients with AS from the baseline to week 12, and significant intergroup differences of additional composite indices of disease activity (i.e., erythrocyte sedimentation rate, patient global assessment of disease activity, total back pain, level of morning stiffness, tender joints, and BASFI scores) were also observed.

**Conclusion:**

Kunxian capsule significantly decreased the disease activity of patients with AS.

**Trial registration:**

NCT00953979. Registered on 5 August 2009.

**Electronic supplementary material:**

The online version of this article (doi:10.1186/s13063-016-1438-6) contains supplementary material, which is available to authorized users.

## Background

Ankylosing spondylitis (AS) is a chronic inflammatory disease that affects the sacroiliac joint, spine bone protrusion, and paraspinal soft tissue and peripheral joints; it may also have extra-articular manifestations. In severe cases, it can cause spinal deformity and ankylosis. Treatment options available for AS are limited. Sulfasalazine (SSZ), which has certain side effects, is one of the most common treatments, and is mainly used in AS patients with peripheral arthritis [1]. TNF-α receptor antagonists are also used, but are very expensive [2]. Therefore, an inexpensive drug with acceptable side effects is needed.

Kunxian capsule’s main component is triptolide [3], which is extracted from the genus Tripterygium plant Tripterygium hypoglaucum (levl.) Hutch. The genus Tripterygium is from the Celastraceae plant family, and different Tripterygium species have similar chemical compositions and clinical efficacy [4]. Tripterygium has been used in 21 autoimmune and inflammatory diseases by rheumatologists in China since 1983 [5] to treat, e.g., rheumatoid arthritis (RA) [6, 7], systemic lupus erythematosus (SLE) [8–10], and nephritis [10–13]. Since 2006, Kunxian capsule has been used in clinical settings as an anti-inflammatory and immune system regulator of RA [14–16], SLE [17], AS [18], and other inflammatory and autoimmune diseases.

Although the therapeutic effect of Kunxian has been investigated in the treatment for patients with AS, the effect has rarely been assessed by international indicators. This study used the Assessment of SpondyloArthritis International Society response rate 20/40/70 (ASAS 20/40/70), Bath Ankylosing Spondylitis Disease Activity Index 20/50/70 (BASDAI 20/50/70), Ankylosing Spondylitis Disease Activity Score on the basis of C-reactive protein level (ASDAS-CRP), Bath Ankylosing Spondylitis Functional Index (BASFI), Bath Ankylosing Spondylitis Metrology Index (BASMI), and other indices to measure the efficacy of Kunxian capsules in the treatment of AS patients.

## Methods

This study was approved by the Ethics Committee of the Third Affiliated Hospital of Sun Yat-sen University and registered at ClinicalTrials.gov with identifier NCT00953979.

### Patients

The patients with AS were those who visited the hospital from December 2008 to February 2010, who were aged from 18 to 65 years and living in China. They were randomly divided into two groups according to a random number table. AS patients who fulfilled the modified New York criteria (1984) and had active disease defined by BASDAI ≥40 mm under background stable nonsteroidal anti-inflammatory drugs (NSAIDs) for more than 4 weeks were enrolled. Patients taking disease-modifying antirheumatic drugs (DMARDs) or biologics within 4 weeks before the study were excluded. Other exclusion criteria included intra-articular injection within 3 months; history of heart failure, multiple sclerosis, chronic obstructive pulmonary disease, recurrent infection, lymphoma, or other cancers; fibromyalgia or other rheumatic diseases; pregnant and lactating women; previous poor compliance, mental illness, and alcohol or drug addiction. The enrolled patients were not allowed to change background NSAID dosage or add other anti-rheumatic drugs during the study. After being informed of the details of the research, all patients or their guardians provided written informed consent to participate.

### Intervention

Macroporous resin adsorption was used to prepare the Kunxian. The preparation, quality control, and standardized testing of Kunxian capsules and the placebo were applied for and approved by China’s State Food and Drug Administration and manufacturer (approval number: YBZ07522006-2009Z; 1514062). In the Kunxian group, two Kunxian capsules (0.3 g/capsule, with a triptolide content of 25 μg/capsule) were taken orally three times a day for 12 weeks.

### Assessment

Patients were examined and evaluated at weeks 0, 2, 6, and 12. Clinical assessment was performed using indices of BASDAI, BASFI, Bath AS Patient Global Score (BAS-G), nocturnal back pain, general back pain, and morning stiffness. Spinal motility was assessed by chest expansion and the Schober test. Laboratory tests for erythrocyte sedimentation rate (ESR), C-reactive protein (CRP), alanine aminotransferase (ALT), aspartate aminotransferase (AST), blood urea nitrogen, and serum creatinine were carried out. Adverse events and concomitant medications were recorded. The primary endpoint of efficacy was the proportion of patients who achieved ASAS 20 according to the Assessment of SpondyloArthritis International Society (ASAS). The secondary endpoints of efficacy were ASAS 40, BASDAI 50, and ASDAS-CRP calculated according to the following formula: ASDAS-CRP = 0.121 × back pain + 0.058 × morning stiffness duration + 0.11 × overall patient discomfort degree + 0.073 × peripheral joint pain or swelling degree + 0.579 × Ln(CRP + 1).

### Statistical analysis

The sample size was calculated based on findings of the previous clinical studies in patients with AS. For the primary endpoint, a sample size of 10 patients per protocol analysis set in each treatment group was required according to 90 % statistical power based on a one-sided test at 0.05. Thus, a target sample size of 40 patients per treatment group was estimated with a drop-out rate of 20 %. Missing data were imputed using the last observation carried forward (LOCF) method. Data were presented as mean ± standard deviation (SD). All analyses were performed by using the Statistical Package for the Social Sciences for Windows (SPSS), version 13.0 and Microsoft Office Excel. Categorical data (such as ASAS 20/40/70 and BASDAI 20/50/70) were expressed as numbers or percentages and analyzed using the chi-square test or Fisher’s exact probabilities. The *t* test was used to investigate the difference in clinical parameters such as BASFI, ASDAS-CRP, BASMI, CRP, and ESR. Any *p* values less than 0.05 were considered statistically significant.

## Results

### Disposition and characteristics of the patients

**Fig. 1:**
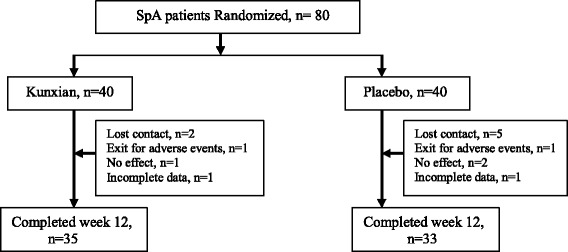
Patient disposition. A total of 80 patients with AS were enrolled, among whom contact was lost in 7 patients (2 patients in Kunxian group and 5 in placebo group), 2 patients exited for adverse events (one in Kunxian group and one in placebo group), and 2 patients had incomplete information (one in Kunxian group and one in placebo group). Finally, 68 patients completed clinical observation throughout the 12 weeks (35 in Kunxian group and 33 in placebo group)

A total of 80 patients with a mean age of 31.9 ± 11.7 years were randomly divided into two groups, the Kunxian group (40 patients) and the placebo group (40 patients) (Fig. 1). Among the 80 patients, 79 (98.78 %) had inflammatory spinal pain. There were no statistical differences in the baseline characteristics between the Kunxian group and the placebo group (Table 1). The distribution of demographical characteristics of the participants is shown in Table 1. No differences between variables were observed.

**Table 1:** Baseline demographics and characteristics of the patients with AS

Information	Kunxian (*n* = 35)	Placebo (*n* = 33)
Demographics		
Male, *n* (%)	32 (91.4)	30 (90.9)
Age, years (SD)	27.19 (7.94)	28.83 (7.09)
Disease characteristics		
Symptom duration in years	6.76 (5.47)	7.98 (4.78)
Concomitant NSAID, *n* (%)	13 (37.1)	15 (45.5)
Clinical features, *n* (%)		
Inflammatory back pain	32 (91.4)	32 (97.0)
Elevated CRP (>3 mg/liter)	31 (88.6)	24 (72.7)
Elevated ESR (male >20; female >15 mm/h)	32 (91.4)	29 (87.9)
Disease activity		
ASDAS-CRP	3.36 (1.06)	3.41 (1.00)
BASDAI (0–10)	4.79 (1.45)	4.82 (1.22)
Total back pain (0–10)	5.09 (2.69)	5.34 (2.58)
Global assessment (0–10)	5.18 (2.08)	5.90 (1.85)
Swollen joints, range 0–44	0.46 (1.48)	0.29 (1.1)
Tender joints, range 0–44	5.57 (4.96)	4.91 (3.37)
BASFI (0–10)	3.10 (2.00)	2.80 (2.00)
Inflammation/morning stiffness (0–10)	4.79 (2.79)	5.2 (2.43)
ESR (mm/h)	36.95 (25.26)	35.79 (31.46)
CRP (mg/liter)	29.5 (29.93)	27.50 (29.69)
BASMI (0–10)	2.88 (1.95)	3.75 (1.99)

All values are expressed as mean (SD) unless otherwise indicated

*Abbreviations*: *NSAID* nonsteroidal anti-inflammatory drugs, *CRP* C-reactive protein, *ESR* erythrocyte sedimentation rate, *ASDAS* Ankylosing Spondylitis Disease Activity Score, *BASDAI* Bath Ankylosing Spondylitis Disease Activity Index, *VAS* visual analog scale, *BASFI* Bath Ankylosing Spondylitis Functional Index, *BASMI* Bath Ankylosing Spondylitis Metrology Index

### Efficacy

**Fig. 2:**
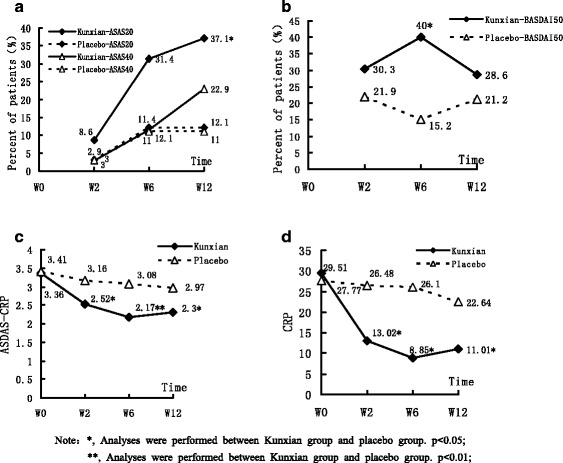
The changes of ASAS 20, BASDAI 50, ASDAS-CRP, and CRP at weeks 0, 2, 6, and 12 in the patients with ankylosing spondylitis treated with Kunxian. **a** ASAS 20 in Kunxian group was significantly higher than that in placebo group at week 12 (*p* < 0.05). **b** BASDAI 50 in Kunxian group was significantly higher than that in placebo group at week 6 (*p* < 0.001). **c** ASDAS-CRP in Kunxian group was significantly higher than that in placebo group, especially at week 6 (*p* < 0.001). **d** The change of serum CRP in Kunxian and placebo groups. Serum CRP in Kunxian group was significantly lower than that in placebo group at weeks 2, 6, and 12 (*p* < 0.05)

The primary endpoint of ASAS 20 at week 12 was achieved in 13 of 35 patients (37.1 %) in the Kunxian group, compared with 4 of 33 (12.1 %) in the placebo group (*p* < 0.05) (Fig. 2a). Significant improvements of BASDAI 50 were also observed in the Kunxian-treated patients at week 6 (14 (40 %) and 5 (15.5 %), respectively, *p* < 0.05) (Fig. 2b). At weeks 2, 6, and 12, the value of ASDAS-CRP in the Kunxian group was significantly lower than that in the placebo group, especially at week 6 (*p* < 0.01) (Fig. 2c), indicating that Kunxian obviously reduced CRP compared to the placebo (*p* < 0.05) at weeks 2, 6, and 12 (Fig. 2d).

Compared with the placebo, Kunxian was associated with significantly greater improvements in signs and symptoms of patients with AS from the baseline to week 12, and significant intergroup differences were also observed in terms of additional composite indices of disease activity (i.e., BASFI, ESR, patient global assessment of disease activity, total back pain, level of morning stiffness, tender joints, and BASMI scores) (Table 2).

**Table 2:** Efficacy at weeks 2, 6, and 12

Activity index	Time	Mean ± SD change from the baseline	
		Kunxian, *n* = 35	Placebo, *n* = 33
BASDAI (0–10), mean (±SD)	Week 2	-1.46 ± 1.61	-0.91 ± 1.31
	Week 6	-1.98 ± 1.48^a^	-0.10 ± 1.13
	Week 12	-1.68 ± 1.70	-1.11 ± 1.17
BASFI (0–10), mean (±SD)	Week 2	-0.52 ± 1.17	-0.15 ± 1.32
	Week 6	-0.88 ± 1.18^c^	-0.28 ± 1.27
	Week 12	-0.75 ± 2.26	-0.01 ± 1.59
ASDAS-CRP, mean (±SD)	Week 2	-0.84 ± 0.91^b^	-0.05 ± 0.98
	Week 6	-1.25 ± 0.95^a^	-0.06 ± 0.70
	Week 12	-1.08 ± 1.03^b^	-0.11 ± 1.79
ESR (mm/h), mean (±SD)	Week 2	-11.26 ± 16.38^c^	0.15 ± 9.48
	Week 6	-16.26 ± 18.63^b^	0.71 ± 16.76
	Week 12	-14.51 ± 22.65^c^	0.09 ± 16.50
CRP (ref 6 mg/l), mean (±SD)	Week 2	-16.49 ± 21.12^b^	0.37 ± 16.55
	Week 6	-20.66 ± 23.71^b^	1.07 ± 20.20
	Week12	-18.50 ± 26.18^c^	0.76 ± 16.67
Level of morning stiffness, 0–10 cm VAS	Week2	-2.56 ± 2.81^c^	-0.88 ± 1.37
	Week6	-2.10 ± 2.39^c^	-0.78 ± 2.38
	Week 12	-2.53 ± 2.60^c^	-1.34 ± 1.67
Global assessment, 0–10 cm VAS	Week 2	-1.29 ± 1.94	-0.54 ± 1.30
	Week 6	-1.99 ± 2.10^c^	-0.81 ± 1.31
	Week 12	-1.69 ± 2.30	-0.85 ± 1.55
Total back pain, 0–10 cm VAS	Week 2	-1.22 ± 1.99	-0.45 ± 1.60
	Week 6	-1.97 ± 2.08^c^	--0.81 ± 1.98
	Week 12	-1.94 ± 2.17^c^	-0.77 ± 1.57
Swollen joints, range 0–44	Week 2	-0.14 ± 0.42	0.14 ± 0.85
	Week 6	-0.16 ± 0.5	-0.05 ± 0.34
	Week 12	0 ± 0.82	-0.06 ± 0.34
Tender joints, range 0–44	Week 2	-1.19 ± 2.77	-0.35 ± 0.73
	Week 6	-2.38 ± 3.66^c^	-0.26 ± 1.24
	Week 12	-2.89 ± 3.43^c^	-0.36 ± 1.32
BASMI (0–10), mean (±SD)	Week 2	-0.32 ± 0.94	-0.32 ± 0.97
	Week 6	-0.49 ± 1.10	-0.14 ± 0.98
	Week 12	-0.24 ± 1.09	-0.21 ± 0.95

^a^Compared with placebo group. *p* **<** 0.0001

^b^Compared with placebo group. *p* **<** 0.001

^c^Compared with placebo group. *p* < 0.05

### Safety

There were 11 patients with adverse events, 8 in the Kunxian group and 3 in the placebo group. The main adverse events were mild to moderate elevation of transaminase, dysmenorrhea, menopause, stomach burning sensation, gastrointestinal discomfort, slightly decreased WBC or platelets, mild urinary tract infection, and dry throat. There was no significant difference in the number of patients between the two groups (Table 3).

**Table 3:** Adverse events during 12-week period of the study

Adverse reactions	AS group (*N* = 68)	
	Kunxian, *n* = 35	Placebo, *n* = 33
Elevated AST/ALT	3	2
Dysmenorrhea	1	0
Gastrointestinal discomfort	4	1
Leukopenia/thrombocytopenia	0	0
Urinary tract infection (urinary frequency, hematuria)	0	0
Dry throat	0	0
Drug allergy	0	0
Total	8^a^	3

^a^Compared with placebo group. *p* > 0.05

We will disseminate the results of our study via presentations at international conferences and publications in peer-reviewed journals. The study will be implemented and reported in line with Standard Protocol Items for Randomised Trials (Additional file 1).

## Discussion

AS is a chronic inflammatory autoimmune disease. The activity of AS reflects the level of inflammation, predicts prognosis, and influences treatment decisions. Currently, the most accepted and widely used indicators to assess the disease activity of AS are ASAS 20/40 and BASDAI [19]. Most studies have utilized these indicators in the clinical research of Kunxian [14, 18]. However, both indicators have been self-administered by patients or physicians—directed questionnaires and objective measures of the disease activity are not included. The whole spectrum of the disease activity of AS is not reflected. The Ankylosing Spondylitis Disease Activity Score on the basis of CRP (ASDAS-CRP) can assess the disease activity and efficacy of different therapies in AS. It has been primarily applied in different races and has shown good clinical value [20–22]. Based on feasibility, the ASDAS consisting of total back pain, duration of morning stiffness, the BASDAI question on peripheral joints, patient global score of disease activity, and CRP were selected as the preferred ASDAS (ASDAS-CRP) [23]. However, in vitro and animal model studies have shown that the genus Tripterygium possesses anti-inflammatory and immunosuppressive properties, including suppression of proinflammatory cytokine production [24, 25]. In this study, Kunxian demonstrated better efficacy than a placebo in suppressing the disease activity of patients with AS as assessed by ASAS 20, BASDAI 50, ASDAS-CRP, and serum CRP as well as patient global assessment of the disease activity, total back pain, level of morning stiffness, tender joints, and BASFI score.

Although Kunxian had good efficacy in patients with AS, and its main component, Tripterygium hypoglaucum (levl.) Hutch, had weaker toxicity and side effects than Tripterygium wilfordii [26], some adverse events were observed in this study, including mild to moderate liver toxicity, decreased WBC or platelets, and gastrointestinal discomfort. These adverse events had also been observed in the treatment of other diseases such as RA [14] and refractory nephrotic syndrome [27]. Many Chinese papers reported that the most severe side effect of Tripterygium was infertility, although animal experiments showed it had no significant effect on infertility or teratogenicity in rats, embryos, or fetuses [28]. Only one case of dysmenorrhea was observed in the study.

Tripterygium hypoglaucum (levl.) Hutch nourishes the kidneys and strengthens the essence. The other three medicines, Epimedium, Dodder, and Lycium Chinense, mainly contain flavonoids. According to the theory of traditional Chinese medicine, Epimedium and Dodder can enhance kidney yang and Lycium Chinense can enhance kidney yin. In China these three medicines have been used for thousands of years. Thus, Epimedium, Lycium Chinense, and Dodder can inhibit the reproductive toxicity of Tripterygium hypoglaucum (levl.) Hutch without affecting its anti-inflammatory and immunosuppressive effects (the theory of Chinese medicine considers that the kidneys store the vital essence and dominate reproduction). Good results were observed in this study, but the impact of Kunxian on human reproductive function remains to be further studied.

## Conclusion

Kunxian capsules have better efficacy than a placebo in decreasing the disease activity of patients with AS assessed by international indicators ASAS 20, BASDAI 50, ASDAS-CRP, and serum CRP as well as by patient global assessment of the disease activity, total back pain, level of morning stiffness, tender joints, and BASFI score.

## Abbreviations

AS, Ankylosing spondylitis; BASDAI 20/50/70, (improvement criteria) Bath Ankylosing Spondylitis Disease Activity Index 20/50/70; NSAID, nonsteroidal anti-inflammatory drug; ASAS 20/40/70, (improvement criteria) the Assessment of SpondyloArthritis international Society 20/40/70 (refer to http://www.asas-group.org/publications/ASAS-handbook.pdf); BASFI, Bath Ankylosing Spondylitis Functional Index; BASMI, Bath Ankylosing Spondylitis Metrology Index; ASDAS-CRP, Ankylosing Spondylitis Disease Activity Score on the basis of C-reactive protein level; SSZ, sulfasalazine; RA, rheumatoid arthritis; SLE, systemic lupus erythematosus; DMARD, disease-modifying antirheumatic drug

## Additional file

Additional file 1: The CONSORT checklist of the trial. (DOC 82 kb)
