# Supplementary material for: Kunxian capsules in the treatment of patients with ankylosing spondylitis: a randomized placebo-controlled clinical trial
Source: Trials. 2016 Jul 22;17:337. doi: 10.1186/s13063-016-1438-6 (PMC4957347; doi:10.1186/s13063-016-1438-6)
Supplement: Additional file 1: — The CONSORT checklist of the trial. (DOC 82 kb) [file 13063_2016_1438_MOESM1_ESM.doc]

| Section/item  **The CONSORT checklist of the trial** | ItemNo | Description | Page |
| --- | --- | --- | --- |
| **Administrative information** | | |  |
| Title | 1 | Descriptive title identifying the study design, population, interventions, and, if applicable, trial acronym | Page 1, line 2-4 |
| Trial registration | 2a | Trial identifier and registry name. If not yet registered, name of intended registry | Page 2, line 75-76 |
|  | 2b | All items from the World Health Organization Trial Registration Data Set | Not applicable |
| Protocol version | 3 | Date and version identifier | Not applicable |
| Funding | 4 | Sources and types of financial, material, and other support | Page 6, line 272-277 |
| Roles and responsibilities | 5a | Names, affiliations, and roles of protocol contributors | Page 6, line 281 |
|  | 5b | Name and contact information for the trial sponsor | Page 6, line 283-285 |
| Introduction |  |  |  |
| Background and rationale | 6a | Description of research question and justification for undertaking the trial, including summary of relevant studies (published and unpublished) examining benefits and harms for each intervention | Page 2, line 36-60 |
|  | 6b | Explanation for choice of comparators | Page 2, line 61-72 |
| Objectives | 7 | Specific objectives or hypotheses | Page 2, line 61-72 |
| Trial design | 8 | Description of trial design including type of trial (eg, parallel group, crossover, factorial, single group), allocation ratio, and framework (eg, superiority, equivalence, noninferiority, exploratory) | Page 2, line 61-72  Page 2, line 80-82  Page 2, line 103-107 |
| Methods: Participants, interventions, and outcomes | | |  |
| Study setting | 9 | Description of study settings (eg, community clinic, academic hospital) and list of countries where data will be collected. Reference to where list of study sites can be obtained | Page, line 78-98 |
| Eligibility criteria | 10 | Inclusion and exclusion criteria for participants. If applicable, eligibility criteria for study centres and individuals who will perform the interventions (eg, surgeons, psychotherapists) | Page, line 78-98 |
| Interventions | 11 | Interventions for each group with sufficient detail to allow replication, including how and when they will be administered | Page 3, line 102-110 |
| Outcomes | 12 | Primary, secondary, and other outcomes, including the specific measurement variable (eg, systolic blood pressure), analysis metric (eg, change from baseline, final value, time to event), method of aggregation (eg, median, proportion), and time point for each outcome. Explanation of the clinical relevance of chosen efficacy and harm outcomes is strongly recommended | Page 3, line 164-167  Table 1  Page 4, line 168-183  Figure 2  Table 2 |
| Participant timeline | 13 | Time schedule of enrolment, interventions (including any run-ins and washouts), assessments, and visits for participants. A schematic diagram is highly recommended (see Figure) | Page3, line 107-110 |
| Sample size | 14 | Estimated number of participants needed to achieve study objectives and how it was determined, including clinical and statistical assumptions supporting any sample size calculations | Page 3, line 132-139 |
| Recruitment | 15 | Strategies for achieving adequate participant enrolment to reach target sample size | Page 3, line 153-163  Figure 1 |
| **Methods:** **Assignment of interventions (for controlled trials)** | | |  |
| Allocation: |  |  |  |
| Sequence generation | 16a | Method of generating the allocation sequence (eg, computer-generated random numbers), and list of any factors for stratification. To reduce predictability of a random sequence, details of any planned restriction (eg, blocking) should be provided in a separate document that is unavailable to those who enrol participants or assign interventions | Page 3, line 153-157 |
| Implementation | 16b | Who will generate the allocation sequence, who will enrol participants, and who will assign participants to interventions | Page 6, 279-285 |
| **Methods: Data collection, management, and analysis** | | |  |
| Data collection methods | 17a | Plans for assessment and collection of outcome, baseline, and other trial data, including any related processes to promote data quality (eg, duplicate measurements, training of assessors) and a description of study instruments (eg, questionnaires, laboratory tests) along with their reliability and validity, if known. Reference to where data collection forms can be found, if not in the protocol | Page 3, line 112-130 |
|  | 17b | Plans to promote participant retention and complete follow-up, including list of any outcome data to be collected for participants who discontinue or deviate from intervention protocols | Page 3, line 139-140 |
| Statistical methods | 18 | Statistical methods for analysing primary and secondary outcomes. Reference to where other details of the statistical analysis plan can be found, if not in the protocol | Page 3, line 141-151 |
| **Methods: Monitoring** | | |  |
| Data monitoring | 19 | Description of any interim analyses and stopping guidelines, including who will have access to these interim results and make the final decision to terminate the trial | Not applicable |
| Harms | 20 | Plans for collecting, assessing, reporting, and managing solicited and spontaneously reported adverse events and other unintended effects of trial interventions or trial conduct | Page 4, line 184-192  Table 3 |
| Auditing | 21 | Frequency and procedures for auditing trial conduct, if any, and whether the process will be independent from investigators and the sponsor | Not applicable |
| Ethics and dissemination | | |  |
| Research ethics approval | 22 | Plans for seeking research ethics committee/institutional review board (REC/IRB) approval | Page 2, line 73-74 |
| Protocol amendments | 23 | Plans for communicating important protocol modifications (eg, changes to eligibility criteria, outcomes, analyses) to relevant parties (eg, investigators, REC/IRBs, trial participants, trial registries, journals, regulators) | Not applicable |
| Consent or assent | 24a | Who will obtain informed consent or assent from potential trial participants or authorised surrogates, and how (see Item 32) | Page 2, line 98-100 |
|  | 24b | Additional consent provisions for collection and use of participant data and biological specimens in ancillary studies, if applicable | Not applicable |
| Declaration of interests | 25 | Financial and other competing interests for principal investigators for the overall trial and each study site | Page 6, line 287 |
| Ancillary and post-trial care | 26 | Provisions, if any, for ancillary and post-trial care, and for compensation to those who suffer harm from trial participation | Not applicable |
| Dissemination policy | 27a | Authorship eligibility guidelines and any intended use of professional writers | Not applicable |
|  | 27b | Plans, if any, for granting public access to the full protocol, participant-level dataset, and statistical code | Not applicable |
